# Smartphone-based ecological momentary assessment and intervention in a coping-focused intervention for hearing voices (SAVVy): study protocol for a pilot randomised controlled trial

**DOI:** 10.1186/s13063-018-2607-6

**Published:** 2018-05-02

**Authors:** Imogen H. Bell, Sarah F. Fielding-Smith, Mark Hayward, Susan L. Rossell, Michelle H. Lim, John Farhall, Neil Thomas

**Affiliations:** 1 Centre for Mental Health, Swinburne University of Technology, Hawthorn, Australia; 2 Monash Alfred Psychiatry Research Centre, Melbourne, Australia; 3 Sussex Partnership NHS Foundation Trust, Worthing, UK; 4 School of Psychology, University of Sussex, Brighton, UK; 5 Department of Psychiatry, St. Vincent’s Hospital, Melbourne, Australia; 6 Department of Psychology and Counselling, La Trobe University, Melbourne, Australia; 7 NorthWestern Mental Health, Melbourne Health, Melbourne, Australia

**Keywords:** Digital technology, Smartphone app, Ecological momentary assessment, Ecological momentary intervention, Psychosis, Hearing voices, Auditory hallucinations, Randomised controlled trial

## Abstract

**Background:**

Smartphone-based ecological momentary assessment and intervention (EMA/I) show promise for enhancing psychological treatments for psychosis. EMA has the potential to improve assessment and formulation of experiences which fluctuate day-to-day, and EMI may be used to prompt use of therapeutic strategies in daily life. The current study is an examination of these capabilities in the context of a brief, coping-focused intervention for distressing voice hearing experiences.

**Methods/design:**

This is a rater-blinded, pilot randomised controlled trial comparing a four-session intervention in conjunction with use of smartphone EMA/I between sessions, versus treatment-as-usual. The recruitment target is 34 participants with persisting and distressing voice hearing experiences, recruited through a Voices Clinic based in Melbourne, Australia, and via wider advertising. Allocation will be made using minimisation procedure, balancing of the frequency of voices between groups. Assessments are completed at baseline and 8 weeks post-baseline. The primary outcomes of this trial will focus on feasibility and acceptability of the intervention and trial methodology, with secondary outcomes examining preliminary clinical effects related to overall voice severity, the emotional and functional impact of the voices, and emotional distress.

**Discussion:**

This study offers a highly novel examination of specific smartphone capabilities and their integration with traditional psychological treatment for distressing voices. Such technology has potential to enhance psychological interventions and promote adaptation to distressing experiences.

**Trial registration:**

Australian New Zealand Clinical Trial Registry, ACTRN12617000348358. Registered on 7 March 2017.

**Electronic supplementary material:**

The online version of this article (10.1186/s13063-018-2607-6) contains supplementary material, which is available to authorized users.

## Background

There have been dramatic advances in digital technology over the last two decades, opening unprecedented opportunities for developing tools to assist in mental health care. These tools include computers, tablets, smartphones and wearable sensors, which can provide both accessible and cost-effective support, as well as the potential for more reliable and effective methods of assessment and monitoring [1]. For those with persisting mental health problems, such as psychosis, where access to support and self-management is often required on a day-to-day basis, these technologies may assist in overcoming existing barriers to effective treatment. In addition, they are usually accessed independently and on demand, and can therefore be empowering to users in self-managing their own condition, a key element of personal recovery [2, 3].

Digital technologies range in their features, designs and platforms, providing opportunities to target different needs associated with mental health conditions. Smartphones in particular have become a strong focus of digital health research due to their capabilities to provide in-situation support in daily life through specialised applications (“apps”) [4]. Smartphone apps are a booming area of both research and industry, with more empirical evaluations needed to help refine features to minimise risks and maximise benefits [5].

One promising use of smartphone apps is assisting with the self-management of psychotic experiences such as hearing voices [6–9]. These experiences, commonly observed as persistent symptoms in psychotic disorders, such as schizophrenia, can be distressing and impairing [10, 11]. Psychological intervention largely involves promoting the ability to adapt to and self-manage these experiences in a way that reduces the impact on the individual’s daily life [12]. Given that voices may be experienced as frequent and unpredictable, the on-demand availability of smartphone apps suggests potential for assisting in this active self-management when it is needed.

Several studies have examined the use of smartphone apps in people with persisting mental health problems, including psychotic conditions. A recent meta-analysis examined ownership of mobile devices and attitudes to their use in mental health care in psychiatric patients [13]. Across 15 studies including a total of 2129 patients with a psychotic disorder, 66.4% of patients owned a mobile phone, which had increased over time to 81.4% between 2013 and 2015; furthermore, the majority of patients reported interest in using mobile devices for health monitoring and tracking, access to information about their health, scheduling appointment reminders and facilitating contact with service providers. Indeed, recent research has shown patients are already frequently using mobile technology for these purposes, as well as assisting with coping with symptoms and connecting with others [14, 15]. Initial trials have indicated evidence that online and mobile technologies are feasible and acceptable for people with psychotic disorders [9, 16, 17].

One important way in which people with psychotic experiences may benefit from smartphone apps is through their ability to prompt adaptive behaviours when they are needed in daily life. This application of mobile technology is termed “ecological momentary intervention” (EMI [18]), involving the delivery of reminders or prompts to users via mobile devices. EMIs have received increased attention in recent years, with evidence for their benefit in a variety of mental and physical health conditions [18–23].

A related use of smartphone technology is termed “ecological momentary assessment” (EMA [24]). EMA is an assessment method involving the regular and frequent self-assessment of momentary experiences in the context of daily life. This method has been used extensively in psychosis research as a means of recording and examining momentary psychotic experiences and their relationship to internal and external variables [25–27]. As assessment is occurring repeatedly, in real time, and in natural environments, there is greater ecological validity, data is less subject to retrospective recall bias, and it is possible to examine the relationship between variables over time. EMA and EMI (EMA/I) can be combined to provide response-dependent intervention strategies via smartphone apps in daily life.

A recent systematic review identified nine studies with broad application of EMA/I in the assessment and treatment of psychotic conditions, ranging from specialised smartphone apps based on cognitive behavioural therapy to remote assessment of symptom states to initiate early intervention for relapse [21]. These studies found the applications to be feasible and acceptable for patients, and there was evidence that they can be used to promote self-management of symptoms and improve functioning [28, 29].

EMA/I may be particularly suited to assisting with the self-management of voice hearing experiences, building on prior therapeutic approaches involving enhancement of coping strategies targeting voices and their triggers [30]. Improving coping with voices is a key component of cognitive behavioural therapy for psychosis [31] and has been formally operationalised in a therapeutic approach developed by Tarrier and colleagues [30] referred to as “Coping Strategy Enhancement” (CSE). CSE involves an initial functional analysis of antecedents and responses to psychotic experiences to inform the identification and implementation of coping strategies in daily life. Research trials and case studies have shown broad support for CSE approaches in treating psychotic experiences, although there are limited examinations of this approach in isolation and targeting voices specifically [30, 32–37].

EMA/I might be used to augment such coping-focused approaches to voices in several ways. First, EMA provides a means of capturing more reliable information about the way in which symptoms interact and evolve over time, which has been shown to be superior to retrospective self-report [26, 38–40]. To aid functional analysis as the first stage of coping interventions for voices, EMA data could be collected, analysed and produced as in-session feedback to identify predictors of fluctuations in voices. Voice hearing has been shown to vary depending on the individual’s internal states (e.g. mood) and external stimuli (e.g. social context) (see [41]). With EMA, contextual variables can be assessed alongside features of the voices themselves, such as intensity/loudness, allowing for the examination of within-person predictors of voice fluctuations (e.g. [25]). Second, it is common for psychological interventions to require clients to recall the evolution of events, which can be challenging for those with psychosis who tend to display cognitive difficulties involving poor recall [42]. EMA shares similarities to typical diary methods used in CBT for this purpose, involving recording of information in monitoring sheets outside of therapy to aid identification of patterns requiring intervention and also to foster self-awareness through self-monitoring. Electronic delivery of reminders and recording of information via EMA could be more convenient, increase likelihood of completion and provide more accurate and sensitive information [43, 44]. Third, EMI has shown to aid recall and promote the use of symptom self-management strategies in psychosis [28, 45], aligning with the primary aim of coping approaches to voices being to improve the use of more effective coping strategies in daily life.

To summarise, interventions which make use of specialised smartphone capabilities such as EMA/I have shown promise in assisting with management of symptoms and improving recovery, and previous research has found EMA/I are feasible and acceptable to people diagnosed with a psychotic disorder. A particular application is to facilitate self-management of persisting and distressing voices, where EMA/I may offer a means of augmenting the effects of these treatments to promote more consistent and adaptive coping. Given this, the main aim of the proposed study is to investigate the application of EMA/I in a coping-focused intervention for voices (Smartphone Assisted coping-focused interVention for Voices: SAVVy). This paper describes a pilot randomised controlled trial (RCT) with the following objectives.

### Objectives

There are three main objectives in this trial. The first objective is to explore whether the intervention is feasible to deliver and acceptable to participants. Feasibility of smartphone-based EMA self-monitoring will be determined by the proportion of participants receiving the intervention who complete at least 33% of the total number of EMA surveys, allowing production of feedback in session. This cut-off was determined based on recommendations from the EMA literature which suggest that participants should complete at least 33% of the total number of EMA surveys to provide a sufficient number of data points for an analysis that is both reliable and representative of a person’s daily experience [46]. The feasibility of the smartphone-based EMI will also be examined through the number of EMI reminders viewed over the course of the intervention and session attendance rates. Acceptability will be assessed by participant ratings of satisfactions with the intervention and its perceived credibility prior to commencing. The second objective is to examine the feasibility of a full-scale trial through recruitment and attrition rates. The third objective is to provide a preliminary estimation of the clinical impact of this intervention alongside treatment-as-usual (TAU) compared to TAU alone on (1) overall severity of voices, (2) emotional and functional impact of voices and (3) overall emotional distress. As a preliminary examination of possible processes of change in these clinical outcomes, we will also examine changes on measures of the participant’s awareness of factors that influence voices day-to-day and experiences of coping, as these are the primary intervention targets which may theoretically lead to a reduction in voice severity and impact.

## Methods/design

## Design

**Fig. 1:**
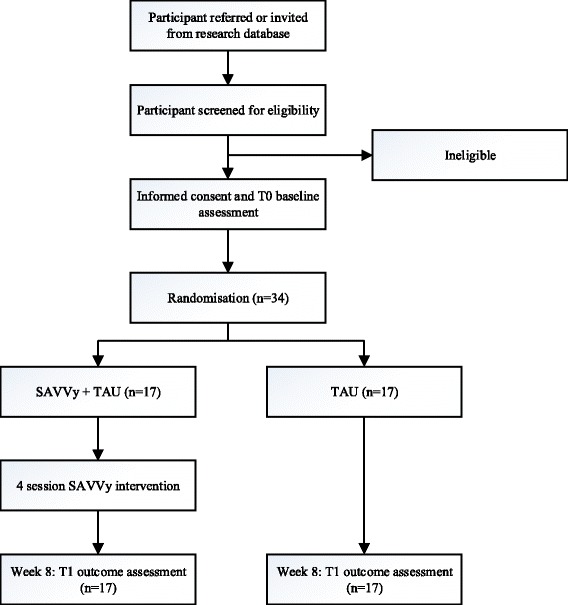
Consolidated Standards of Reporting Trials (CONSORT) diagram showing study design

The SAVVy trial is a pilot randomised, controlled, assessor-blinded trial, with two parallel groups, using a 1:1 allocation ratio. Each consenting participant will be randomised to either SAVVy + TAU or TAU alone, following the informed consent and baseline assessment session. TAU will involve continuation of standard treatment provided by the usual care team (usually consisting of medication and case management without psychological treatment). Baseline assessments will occur pre-randomisation (T0) and at a standard period of approximately 8 weeks following randomisation (T1). All participants will be invited to complete the outcome assessment regardless of whether they complete the full course of intervention. Figure 1 displays the Consolidated Standards of Reporting Trials (CONSORT) flow diagram of the study procedure. The protocol was designed in accordance with the Standard Protocol Items: Recommendations for Interventional Trials (SPIRIT) (Additional file 1) and Good Clinical Practice guidelines.

### Setting

This trial will be conducted at a specialist psychological treatment and research clinic for people who experience persisting and distressing voices based in Melbourne, Australia [47]. The clinic receives referrals from across Melbourne and surrounding areas and offers specialist consultation and psychological interventions for auditory hallucinations.

### Participants

As this is a pilot RCT, power calculations based on effect sizes are not appropriate to inform recruitment targets. Rather, a sufficient sample size is needed for estimating the standard deviation of the outcome measures to inform future power calculations, to determine recruitment and attrition rates, and to evaluate the feasibility and acceptability of the intervention. There is variability across the literature regarding sample size recommendations for pilot RCTs, ranging from 24 to 70 [48–51]. We determined a sample size of 34 (split evenly between groups) would be sufficient to meet the aims of this study, which is within the recommended range and in line with pilot trials of similar interventions (e.g. [28, 52–55]).

Participants meeting the following criteria will be eligible for the study: (1) over the age of 18 years; (2) sufficient conversational English for meaningful participation in the study; (3) experiencing current and persisting auditory verbal hallucinations defined by, over the week preceding the baseline assessment, hearing a hallucinated voice or voices on at least four different occasions *and/or* on at least one occasion and of at least an hour’s duration; we determined this criterion would be the minimum frequency in order to capture their occurrence during the EMA monitoring period; (4) experiencing distress due to the voices (score of 1 or more on item 8 of the Psychotic Symptom Rating Scales-Auditory Hallucinations (PSYRATS-AH [56])); (5) experiencing voices for at least 6 months; and (6) comfortable using a smartphone or willing to learn. Exclusion criteria are as follows: (1) unable to provide informed consent; (2) intellectual disability as assessed by the Wechsler Test of Adult Reading (WTAR [57]) estimated IQ < 70]; (3) voices occur solely in the context of substance or alcohol use; (4) initiation of a new antipsychotic medication within the previous 8 weeks; (5) too distressed or agitated to take part in the study assessed by clinician observation during screening or baseline assessment; (6) current risk of harm to self or others that requires active crisis management.

### Recruitment and enrolment

Recruitment will take place at the Voices Clinic via direct referrals to the study from relevant mental health services, and wider advertising. All clients from the Voices Clinic likely to meet inclusion criteria for the study will be informed of the trial following their initial consultation appointment, alongside other treatment options.

Clients interested in taking part in the trial will then be contacted by a member of the research team independent from their clinical care who will explain the study in more detail and formally screen over the phone for eligibility. Potentially eligible and interested candidates will then be sent participant information consent forms and allowed at least 48 h to consider their participation. Those interested in participating will then be contacted to book a time for the first assessment session, during which eligibility is confirmed. Persons who participate in the trial continue their routine treatment whilst participating in the trial and are able to receive other psychological therapies at the Voices Clinic following the outcome assessment (or withdrawal from the trial), irrespective of condition.

Advertising will also take place at relevant mental health organisations and institutions, and online. Potential participants and mental health professionals will therefore be able to contact the researchers directly if they or a client is interested in participating in the trial. Further, individuals who have previously taken part in research projects at the Voices Clinic and have consented to be contacted about new research opportunities will be offered to take part.

Participants are reimbursed for time attending the two assessment sessions and loaned a smartphone with data allowance for the duration of the trial if they are not able to use their own.

### Allocation and blinding

Participants confirmed to be eligible following the baseline assessment will then be subject to random allocation to groups using minimisation procedure, conducted by an experienced researcher independent of the research team using QMinim online software [58]. Randomisation using minimisation will be used as it allows for better balance of patient characteristics across groups, particularly in trials with small sample sizes [59]. In this trial, minimisation will balance whether voices are experienced as non-continuous or continuous (PSYRATS-AH item 1 score ≤ 3 versus 4), because this covariate is predicted to have an effect on treatment response and should therefore be balanced across groups. Allocation will occur within 2 days of the baseline assessment. Participants will then be contacted to alert them to their allocation, with those allocated to the treatment group commencing therapy within 2 weeks of the baseline assessment and those allocated to the control group confirming a date for the outcome assessment.

All outcome assessments will be conducted by a researcher blind to treatment allocation throughout the study. It is not possible to blind participants to their allocation due to the nature of the trial design. Following allocation to the treatment or control arm, and prior to the outcome assessment, participants will be asked not to reveal their group allocation to the researcher conducting the outcome assessment. Outside of the assessments, researchers conducting the outcome assessments are shielded from discussion of participants, and assessments are conducted on different days to the therapy sessions. In the event of an unblinding during the assessment, this will be recorded and addressed by repeating the clinical interview as soon as possible by another blind assessor. Success of blinding will be assessed by asking the assessor to guess which condition the participant was allocated to and indicate their confidence. The PI (NT) is not blinded and responds to any clinical and research issues during the trial that require knowledge of a participant’s condition.

### Materials

See Table 1 for the SPIRIT schedule of measures used in this study. The primary outcomes will focus on feasibility and acceptability of the intervention and trial methodology, with secondary outcomes evaluating clinical measures. Trained raters will be supervised throughout the study to ensure reliability and validity of the clinical interviews.

**Table 1:** SPIRIT schedule of enrolment, interventions and assessments

	Enrolment	Intervention	Close-out
Timepoint	T0	0–8 weeks	T1
Enrolment			
Eligibility screen	X		
Informed consent	X		
Allocation	X		
Interventions			
SAVVy + TAU		X	
TAU		X	
Assessments			
PSYRATS-AH	X		X
SEPS Negative Impact	X		X
DASS-21	X		X
4-item coping with voices	X		X
Receipt of other treatments	X		X
Medications and dosages	X		X
M.I.N.I. and SCID-5 BPD	X		
SANS	X		
WTAR	X		
Internet use and familiarity questionnaire	X		
CEQ	X		
Demographics	X		
WAI-SR		X	
Satisfaction questionnaire		X	

Demographic variables will include gender, employment, education, country of birth and ethnicity, in addition to medication doses and receipt of other treatments during enrolment. The following measures will also be used to describe the sample.

#### Diagnosis

Two structured clinical interviews will be used to ascertain potential current diagnoses: the Mini-International Neuropsychiatric Interview (M.I.N.I. 7.0 [60]) for DSM-5 mental disorders and the Structured Clinical Interview for DSM-5 (SCID-5 [61]) Borderline Personality Disorder (BPD) section only. The prevalence of voice hearing is more common in BPD compared to other personality disorders [62]; therefore, screening beyond BPD was considered unjustified given the added length of time this would necessitate.

#### Cognitive ability

The Wechsler Test of Adult Reading (WTAR [57]) will be used to estimate premorbid intellectual ability. WTAR raw scores are standardised based on age and then converted to the predicted WAIS-III IQ.

#### Negative symptoms

The Scale for the Assessment of Negative Symptoms (SANS [63]) will be used to measure the severity of negative symptoms of psychosis at baseline. This measure has been included as participants with greater levels of negative symptoms may benefit less from psychological interventions for voices [12]. The SANS is a semi-structured clinical interview which assesses negative symptoms of psychosis across five domains: affective flattening or blunting, alogia, avolution-apathy, anhedonia-asociality, attention. Each of the five symptoms are rated from 0 (*absent*) to 5 (*severe*) based on responses to interview questions and observations made by the assessor, with a total score derived from summing all item scores. Higher scores indicate a greater severity of negative symptoms. The SANS is a very commonly used tool in research for this purpose and has demonstrated good reliability and validity [63, 64].

#### Internet use and familiarity

A seven-item questionnaire will be used to describe the sample in terms of use and comfort with smartphones and the internet in general. An example item is “How confident are you in using applications (“apps”) on a mobile phone?” rated on a 7-point scale from 1 (*not at all confident*) to 7 (*very confident*).

### Feasibility

Assessment of feasibility will focus on the following: completion rates of the EMA surveys (*completers* defined as having completed over 33% of the total number of EMA surveys); the proportion of participants for whom it was possible to produce EMA-based feedback summaries; the number of EMI reminders viewed; recruitment rate (number of participants referred and screened, and proportion of eligible participants consenting to participate) and attrition (proportion of those completing the outcome assessment, and all four sessions of the therapy if allocated to the treatment group); fidelity to the intervention protocol (proportion of therapy checklist items endorsed by therapists as completed).

### Acceptability outcomes

Acceptability will be measured using a specifically designed, 14-item satisfaction questionnaire. All except two items are statements rated on a 5-point Likert scale from 1 (*strongly disagree*) to 5 (*strongly agree*). Example items include “The coping strategy reminders were useful to help me cope with my voices” and “It was useful to discuss the smartphone feedback in therapy”. The item “I would recommend this intervention to other people with voice hearing experiences” will be the primary indicator of intervention acceptability. Open-ended questions will be included to allow participants to provide feedback according to their own subjective experience. In addition, a sample of approximately 12 participants who complete the intervention will be invited to participate in a qualitative interview, which will be reported upon separately to the main RCT. This interview will focus on examining participants’ experiences of different aspects of the intervention and as a whole.

The Credibility/Expectancy Questionnaire (CEQ [65]) will be used to measure a participant’s expectations of the likely success of the intervention and its perceived credibility prior to commencing. This measure is included based on research emphasising participants’ perceptions of the acceptability of the intervention prior to participating are a core element of acceptability and should be evaluated at the feasibility and pilot phases of new interventions [66]. The scale contains six items in total, three referring to the credibility of the intervention (factor 1) and three referring to expectations (factor 2). The CEQ uses two rating scales, one from 1 (*not at all*) to 9 (*very much*) and another from 0% (*not at all*) to 100% (*very much*), with higher total scores indicating a greater expectation of positive effects of the intervention and its perceived credibility. The questionnaire has shown good reliability and validity [65]. The questionnaire will be administered immediately following informed consent procedures during the baseline assessment when the participant will be familiar with the details of the intervention.

The Working Alliance Inventory – Short Revised (WAI-SR [67]) will also be administered to participants in the intervention arm at the end of the final session to provide data on the therapeutic alliance with the therapist. This is a widely used measure of therapeutic alliance between client and practitioner, which corresponds to the extent to which the participant perceives successful alignment on the goals and tasks of therapy, and the accompanying therapeutic bond. The scale contains 12 items covering subscales of goals, tasks and bond between client and therapist, scored on a 5-point scale from 1(*seldom*) to 5(*always*), with some reverse-rated items.

### Clinical outcomes

The following clinical measures will be evaluated as secondary outcomes in this trial to provide a preliminary estimate of efficacy. All measures will request ratings relevant to the past week.

#### Overall severity of voices

The PSYRATS-AH [56] total score will be the primary clinical outcome measure for this trial, reflecting changes in voice phenomenology, severity and impact. The measure is a structured interview examining 11 dimensions of auditory hallucinations, each rated on a 5-point ordinal scale with total scores summed, with higher scores representing greater overall severity of voice hearing experiences. The scale has demonstrated good reliability and validity with sensitivity to change [56, 68].

#### Negative impact of voices

The Subjective Experiences of Psychosis Scale – Negative Impact Subscale (SEPS [69]) will be used to evaluate the negative emotional and functional impact of the voices on the person. The SEPS is a 45-item self-report scale consisting of three subscales assessing experiences of psychosis. In this case, participants will be asked to select “voices” as the psychotic experience they are rating. The total score of subscale 1 “negative impacts of experiences” (29 items) will be used in the current study. Items are rated on a 5-point scale from 1 (*not at all*) to 5 (*very much*), with scores summed to produce an overall score, with higher scores representing a greater negative impact of the voices on the person. The SEPS has demonstrated good to excellent reliability and validity [69].

#### Emotional distress

The Depression, Anxiety and Stress Scale-21 (DASS-21 [70]) scale is a commonly used measure of depression, anxiety and stress. Each of the 21 items is rated on a 4-point scale from 0 (*did not apply to me at all*) to 3 (*applied to me very much, or most of the time*). Subscale scores are totalled and then doubled to yield an overall score, with higher scores indicating a greater level of overall emotional distress. The scale has demonstrated good reliability and validity in clinical samples [71].

### Process measures

Although the sample size and design preclude mediation analysis, we are including several possible process measures in this trial to evaluate their sensitivity to change. These include two single items using a 0–100-mm visual analogue scale (VAS) measuring confidence in coping with the voices and awareness of factors influencing their fluctuations, and two multiple choice items measuring the range of helpful coping strategies and the consistency of their use. These items reflect the primary intervention aims which may theoretically account for reductions in voice severity and impact.

- “How confident are you in your ability to cope with the voice/s day to day?”, VAS anchored 0 mm (*not at all confident*) to 100 mm (*very confident*)
- “I am aware of things which make the voice/s more or less intense”, VAS anchored 0 mm (*not at all*) to 100 mm (*very much*)
- “Over the past week, how often have you used coping strategies for the voice/s?”, multiple choice: 1 (*never when I heard voice/s*); 2 (*a few of the times I heard voice/s*); 3 (*about half of the times I heard voice/s*); 4 (*most times I heard voice/s*); 5 (*every time I heard voice/s*)
- “How many helpful coping strategies do you have to manage the voice/s?”, multiple choice: 1 (*I don’t have any helpful coping strategies for the voice/s*); 2 (*I have one or two helpful coping strategies for the voice/*s); 3 (*I have a few different helpful coping strategies for the voice/s*); 4 (*I have several helpful coping strategies for the voice/s*); 5 (*I have a lot of helpful coping strategies for the voice/s*)

### Planned intervention

**Fig. 2:**
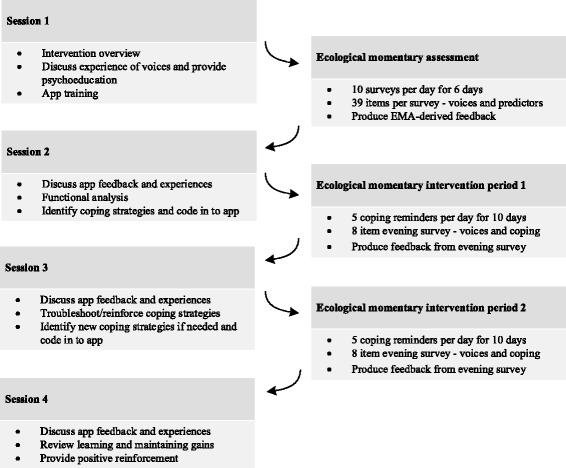
Intervention overview

Participants allocated to the therapy group will receive four face-to-face sessions of approximately 1 h in length, over the 8-week period between baseline and outcome assessments. Sessions are spaced approximately 1–2 weeks apart. In addition, these participants will use a smartphone app between sessions (described below), either their own or one provided to them for the duration of the trial. A breakdown of the intervention details and sequence are provided in Fig. 2, both within and between sessions. The intervention is manualised and will be delivered by the trial coordinator (IB), a trainee psychologist undertaking doctoral studies in clinical psychology, with regular supervision by the PI (NT).

This intervention has been informed by the framework of coping strategy enhancement (CSE [30, 72] and literature on antecedents and coping with voices (e.g. [41, 73–77]). CSE has recently been applied in a brief format targeting distressing voices specifically, supporting feasibility, acceptability and preliminary evidence for improved clinical outcomes [37]. A panel of four individuals with lived experience of voice hearing based in Sussex, UK, were also consulted in the early stages of this research to provide feedback on the design of the intervention and trial, including the wording and schedule of EMA/I surveys.

As an initial phase of the intervention across the first and second sessions, functional analysis will be used to formulate patterns in voice activity, focusing on identifying the antecedents and responses which may give insight into how voices are being maintained. Building from this understanding, the remainder of the intervention will focus on identifying and implementing alternative responses to the voices and their antecedents which may disrupt problematic maintenance cycles associated voice activity and related distress. The therapy can therefore be conceptualised as having two sequential stages: the first involving information gathering and building understanding of daily patterns in voices through functional analysis, and the second involving identification and implementation of individualised coping responses to voices based on the functional analysis. Across these phases, EMA/I will be used to enhance the core aims of the intervention.

#### Stage 1: functional analysis and ecological momentary assessment

Following the first session, participants will complete a 6-day EMA monitoring period during which they complete 10 EMA surveys per day, occurring at semi-random intervals within an individualised 12-h waking period. Each EMA survey will be identical and contain 39 items measuring common antecedents and responses to voices, in addition to voice intensity, distress and impact. A full list of these items and instructions is provided in Additional file 2. The development of the EMA schedule and items involved several iterative stages, including comprehensive literature reviews, consultations with expert clinicians, researchers and lived experience consultants, piloting by researchers, and a small pilot of the intervention with three participants to refine protocols and confirm acceptability of the EMA/I procedure. The purpose of this EMA monitoring period is to provide data which can be analysed to identify potential predictors of voice intensity. In line with recommendations in the literature, at least 33% of the total number of EMA surveys will need to have been completed by the participant in order for analysis of feedback to be conducted [46]. Analysis of the EMA data (produced for the purpose of feedback) will include within-person summary statistics and exploratory multiple regressions conducted on Stata Statistical Software [78], allowing identification of items which predict the intensity of voices. Variables with a standardised beta co-efficient of greater than 0.3 were considered to justify the provision of feedback.

If possible, a feedback sheet will be provided to participants in the second session which will contain the following information: (a) the number of survey responses entered during the monitoring period; (b) average voice intensity and distress scores over the monitoring period; (c) situations, triggers and responses which predicted increased or decreased voice intensity according to multiple regression analysis; and (d) a list of the specific responses to voices reported over the monitoring period and their average reported helpfulness. This feedback will be framed as a guide due to potential limitations in capturing sufficient voice hearing occasions to detect predictors and to avoid inferring causation from correlation data. The feedback will be discussed in the session alongside patterns noticed by the participant during the EMA monitoring period, with the aim of building a functional analysis of voice activity.

### Stage 2: identification and implementation of coping strategies and ecological momentary intervention

Based on the functional analysis conducted across sessions 1 and 2, the therapist and participant will work collaboratively to identify potential coping strategies which may assist the person to cope with their voices and potentially modify patterns in voice activity. Towards the end of the second session, coping strategies will then be chosen by the participant and then coded into the app as personalised coping statements (e.g. “Listen to some relaxing music”). Then, for 10 days following the second session, participants will be sent EMI prompts via the smartphone app of their personalised coping statements. These reminders will occur five times per day, presented semi-randomly within their waking hours. Participants will also be able to view them *on demand* by pressing a button within the app. In addition, participants will complete a short survey each evening asking them about their experiences using the app, the helpfulness of their coping strategies and potential barriers to coping. The latter information is then summarised as feedback and provided to the participant in the following session if possible, with the aim of tracking progress. Based on this discussion, there will be an option to create new coping statements following session 3 or keep the same for a second, identical EMI period. The fourth session will similarly involve reviewing progress and discussing feedback from the app if available, ending with a discussion of how to maintain gains following the end of the intervention.

Fidelity to the intervention protocol will be supported through (a) use of an intervention manual clearly detailing the protocol for each session and procedures between sessions; (b) provision of a participant guide for use during the session which can be used to structure the content and record information; (c) regular supervision of the therapist by the PI, who has expertise in both interventions for voices and the use of digital interventions; and (d) completion of a fidelity checklist following each session by the therapist to record key components of the session.

### Smartphone application

The movisensXS [79] app will be used to facilitate EMA/I in the current study. This app connects with a Web-based platform that allows researchers to program simple and secure EMA/I surveys or prompts that can be individualised for each participant, and download data remotely. The app is downloaded onto the smartphone at the end of the first session, with a schedule of EMA surveys pre-programmed to commence on the following day. At the end of each subsequent session, the app is *updated* (via the Web-based platform) with the participant’s coping strategy reminders, which commence the following day. Use of this survey format was tested by three individuals with persisting voices prior, providing a basis for evaluating the procedures and refining the EMA/I content and design prior to the commencement of the study. Piloting of the movisensXS app was also conducted by the researchers over 7 days to ensure it is reliable for the study purposes.

### Planned analysis

Feasibility of the intervention will be reported as descriptive statistics, focusing on the proportion of participants completing at least 33% of the total number of EMA surveys, the proportion of those in which feedback summaries were able to be produced, and the proportion of EMI reminders viewed. Acceptability will be summarised as means and standard deviations of responses to each item within the satisfaction questionnaire, the proportion of participants giving favourable responses, and representative samples of feedback from open-ended questions. The mean and standard deviation of total scores of the CEQ will also be reported. Feasibility of the trial will be reported descriptively and in figures of referral, screening, consent and attrition, as well as the proportion of fidelity checklist items endorsed as completed. Clinical outcomes will be analysed on an intention-to-treat basis using all available data, adopting statistical approaches to managing missing data if appropriate to the dataset. Results will be summarised as means and/or medians (as appropriate), standard deviations and ranges of all outcome and process measures at T0 and T1 for both groups. Analysis of covariance will be used to estimate T1 between-group differences on all outcome and process measures, controlling for baseline scores. Presentation of the analysis will focus on point estimates and associated 95% confidence intervals rather than statistical significance (*p* values). Standardised effect sizes will be derived using Cohen’s *d*. Secondary analyses will include per-protocol analyses (including only participants who attended at least 50% of therapy sessions) and analyses controlling for baseline moderator variables.

### Data monitoring and management

#### Assessment and therapy data

Training on the administration and scoring of assessments will be provided to all researchers conducting assessments, as well as relevant ethics procedures and protocols for managing and storing data. Regular supervision will also be provided with a continual focus on assessments and data management. Data collected during assessments will be recorded on paper case report forms (CRFs) and securely stored. Data will be extracted to a secure data file by a researcher blind to participant group allocation. For each stage of data cleaning and analysis that is performed, a separate time-stamped computer file will be created and saved within an organised file system. Research data held electronically will be backed up regularly and stored on secure networks. To aid data quality, checks will include examination of recorded data for out-of-range values and examination of a random sample of CRFs by a second researcher to identify potential data entry errors. Files containing treatment information will be kept in a separate location to the assessment files to prevent unblinding and will be extracted to separate electronic data files not accessible to blinded researchers. A formal data monitoring committee is not required by local ethics guidelines for a trial of this type, and no interim analysis is anticipated.

#### Smartphone app data

The movisensXS [79] smartphone app used in the current study is an existing research and clinical tool. The app has been used extensively in EMA research and has been designed to be fully compliant with relevant Australian data protection and health records legislation. No personal identifying information is ever collected within the app. Surveys completed within the app throughout the duration of the treatment are sent via encrypted transfer to a secure online server accessible only by the research team using a secure, registered, password-protected account. The login details will only be known to members of the research team. Data is downloaded and saved in a re-identifiable Excel spreadsheet format, which is then password protected and stored securely.

### Research governance and ethics

The trial is administered by Swinburne University of Technology. This study has been approved by Alfred Hospital Ethics Committee (project 440/16) and Swinburne University Human Research Ethics Committee (project 2016/285). It is conducted in accordance with the Declaration of Helsinki, Good Clinical Practice guidelines and the Australian National Statement on Ethical Conduct in Human Research [80]. Researchers obtain full informed consent from all participants prior to completing the baseline assessment. A copy of the consent form can be requested by contacting the corresponding author. There are no restrictions on reporting findings of this trial, which will be published in full in the peer-reviewed literature. Major protocol amendments will be submitted to ethics committees and detailed in the trial registry and trial protocol if necessary.

### Serious adverse events

Serious adverse events will be monitored and recorded for all enrolled participants throughout the running of the trial. In line with the National Statement on Ethical Conduct in Human Research [80], in the context of this trial, serious adverse events include events that lead to participant death, or that are life-threatening, require inpatient hospitalisation, or result in persistent or significant disability/incapacity. Any such events will be recorded and reviewed with the PI to determine the likelihood of any relationship to the intervention, with action taken as appropriate and notification to the approving Ethics Committees.

## Discussion

The use of digital technology for promoting mental health self-management is becoming a prominent area of research. Smartphone-based ecological momentary assessment and intervention (EMA/I [18, 24]) are particularly promising given their ability to provide momentary assessment of relevant clinical processes and promote adaptive responses to symptoms in daily life. Few research trials have investigated the clinical utility of EMA/I in the form of specialised smartphone apps or mobile devices; however, the existing literature highlights the promise of this technology for promoting self-management of mental health [18–21, 81].

In the context of those with distressing voice hearing experiences, the use of smartphone-based EMA/I may build on existing therapeutic approaches to improving coping with voices. EMA/I may provide important linkages between traditional face-to-face therapy and daily life not only to establish more consistent application of coping strategies discussed in sessions, but also to capture important information often lost in the complexity of daily life. The proposed study is an investigation of such capabilities through evaluation of the feasibility, acceptability and preliminary clinical outcomes of a novel intervention involving smartphone-based EMA/I to improve coping with distressing voices.

There are currently strikingly few empirical evaluations of smartphone apps for mental health [4]. This is problematic given the number of apps readily available for use by consumers, without sufficient understanding of their potentially harmful effects. From another angle, it is critical to understand how these technologies should be designed to maximise their benefits and use across different contexts. The current study aims to evaluate EMA/I as specific smartphone technologies for promoting adaptation to distressing psychotic experiences. The focus on voice hearing experiences was chosen given the logical mapping of EMA/I onto existing therapeutic approaches to coping with voices; however, we hope these findings will provide insights into the use of EMA/I in clinical practice with broader clinical implications.

### Trial status

The trial commenced recruitment with the first participant allocated in March 2017. It is anticipated that the trial will cease recruitment in early to mid-2018.

## Additional files

Additional file 1: SPIRIT 2013 checklist: recommended items to address in a clinical trial protocol and related documents. (DOC 101 kb)

Additional file 2: Smartphone-based EMA and EMI items. (DOCX 26 kb)

## Abbreviations

Apps: mobile phone software applications
CEQ: Credibility/Expectancy Questionnaire
CONSORT: Consolidated Standards of Reporting Trials
CRF: case report form
CSE: Coping strategy enhancement
DASS-21: Depression, Anxiety and Stress Scale-21
EMA: Ecological momentary assessment
EMI: Ecological momentary intervention
M.I.N.I.: Mini-International Neuropsychiatric Interview
PI: Principle investigator
PSYRATS-AH: Psychotic Symptom Rating Scale-Auditory Hallucinations
RCT: randomised controlled trial
SANS: Scale for the Assessment of Negative Symptoms
SAVVy: Smartphone Assisted coping-focused interVention for Voices
SCID-5: Structured Clinical Interview for DSM-5
SEPS: Subjective Experiences of Psychosis Scale
SPIRIT: Standard Protocol Items: Recommendations for Interventional Trials
TAU: Treatment-as-usual
VAS: Visual analogue scale
WAIS-III IQ: Wechsler Adult Intelligence Scale - Third Edition intelligence quotient
WAI-SR: Working Alliance Inventory – Short Revised
WTAR: Wechsler Test of Adult Reading
