# Supplementary material for: Smartphone-based ecological momentary assessment and intervention in a coping-focused intervention for hearing voices (SAVVy): study protocol for a pilot randomised controlled trial
Source: Trials. 2018 May 2;19:262. doi: 10.1186/s13063-018-2607-6 (PMC5930938; doi:10.1186/s13063-018-2607-6)
Supplement: Supplementary file 2 — Smartphone-based EMA and EMI items. (DOCX 26 kb) [file 13063_2018_2607_MOESM2_ESM.docx]

**Additional file 2: Smartphone-based EMA and EMI items**

All EMA items are rated on a 7-point Likert scale (*0-not at all; 3-moderately; 6-very much*) unless stated otherwise. Labels will be displayed only for the end- and mid-point response options (i.e. 0, 3 and 6), with the remaining response options displayed as numbers. Items will be presented one at a time on the screen, and will advance automatically when an answer is selected using the phone touchscreen; as such, the questionnaire is expected to take approximately two-three minutes to complete.

The smartphone app uses conditional branching to automatically advance to a specific subset of items based on a participant’s response. For example, if a participant reports that they are currently hearing voices, the app will *automatically* present relevant voice-related follow-up questions, which differ slightly from the subset of items presented in moments when voices are not reported. In either situation, the number of items presented has been balanced so that the questionnaire takes the same amount of time to complete whether voices are present or absent.

**EMA Daytime Questionnaire**

This 39-item questionnaire will be presented on the smartphone touchscreen ten times per day for a period of six days during the first week of the SAVVy intervention.

**Instructions**

You will now be shown short questions/statements. Please follow the instructions carefully.

**Section A**

Right before the beep…

1. …where were you?

[AT HOME; AT WORK; AT SCHOOL; IN A PUBLIC PLACE; OTHER]

1. …what were you doing?

[NOTHING; SELF-CARE; WORK/STUDY; LEISURE; HEALTH CARE; TRAVEL; OTHER]

1. …how many people were you with?

[0,1,2,3,4+], If 1+, then:

1. I am comfortable with the person/people I am with.

**Section B**

*Right before the beep, I felt:*

1. …happy
2. …satisfied
3. …cheerful
4. …down
5. …lonely
6. …insecure
7. …anxious
8. …stressed
9. …relaxed
10. …that someone was spying on me or plotting against me
11. …suspicious

*Right before the beep…*

1. …my surroundings were noisy

*Right before the beep, I was…*

1. …doing something I’m interested in
2. …doing something important to me
3. …going over problems in my mind
4. …worrying about something
5. …focused on what was going on around me
6. … focused on what I was doing

**___________________________________________________________________________**

**SECTION C**

*Right before the beep…*

1. … I could hear voice/s that other people couldn’t hear

**IF +1,** then continue through section C, then E.

**IF 0,** then continue to section D, then E

1. … It was hard for me to do something because of the voice/s
2. … I was distracted by the voice/s
3. ... the voice/s were distressing me

**___________________________________________________________________________**

**SECTION D**

*Right before the beep, I was…*

1. …felt relieved that I could not hear the voice/s
2. … felt that the voice/s was/were still there, just keeping quiet
3. …was worrying that the voice/s would come back

___________________________________________________________________________

**SECTION E**

**Instructions**

You’ll now see a list of things people sometimes do when they hear voices.

When you see each one, please say whether you a) have done this between the current and previous beep; or b) have not done this

1. Going somewhere quiet.
2. Doing things to help me relax or feel better
3. Sleeping.
4. Drinking, smoking marijuana, or taking illicit substances.
5. Listening to music.
6. Speaking or singing out loud or in your head.
7. Talking to others.
8. Listening to the voice/s.
9. Talking to the voice/s.
10. Arguing with the voice/s (aloud or in head).
11. Doing what the voice/s tell me to do.
12. Ignoring the voice/s.
13. Noticing the voice/s without responding.
14. Thinking things to help me stay calm.

**IF RATED (a) OR (b) TO THE ABOVE:**

1. This response was helpful in managing the voices [presented after each endorsed item, return to previous rating scale]

___________________________________________________________________________

**SECTION F**

***EMA Evening Questionnaire***

The following questions will be presented on the smartphone touch screen within the last waking hour of the participant’s day, each day for the initial 6 day EMA monitoring period. The items are rated on a 7-point Likert scale *(1-strongly disagree; 4- neither agree nor disagree; 7-strongly agree).*

**Instructions**

Please answer each of the following question about how you’ve been doing today.

1. Today, I have felt distressed by the voice/s
2. Today, it was hard for me to do things because of the voice/s
3. Today, I was distracted by the voice/s
4. Today, I noticed some things that made the voice/s better or worse
5. Today, I did things to try to make the voice/s better
6. Today, I have been coping with the voice/s
7. Today, I have been capable of managing the voice/s

**EMI Items**

The EMI period begins after the second session and continues until the fourth session. There are four separate aspects to the smartphone EMI component: 1) morning reminder; 2) five daily reminders of coping strategies; 3) coping strategy reminders initiated by the participant; 4) evening questionnaire.

***Morning reminders***

The following questions will be presented on the smartphone touch screen within the first waking hour of the participant’s day, each day between each intervention session:

1. Good morning! If your voices bother you today, use the app to remind you of your coping strategies.
2. We recommend using the app up to five times a day, but feel free to use it as often as you like!
3. If you are having trouble using the app, take a look at the help guide or give the research team a call.

***Daily coping strategy reminders***

The following instructions are examples of what might be presented on the smartphone touch screen when the participant receives a notification (5 times a day) or opens the app themselves. The wording will be determined by the participant and the therapist in the prior session:

1. You chose the following coping strategies to help you cope with the voice/s. Give them a go if the voice/s are bothering you!
2. Relax! For two minutes, breath in and out slowly, focusing on your breath.
3. Listen! Grab your headphones and listen to some of your favourite music.
4. Tune in to the world! Notice five things around you that you weren’t aware of.

***Evening EMA items***

The following questions will be presented on the smartphone touch screen within the last waking hour of the participant’s day, each day between each intervention session. Statement items are rated on a 7-point Likert scale *(0-not at all; 3-moderately; 6-very much).*

*Use of coping strategies*

1. Did you use any of the following coping strategies today to cope with the voice/s?
   1. Relax! For two minutes, breath in and out slowly, focusing on your breath.
   2. Listen! Grab your headphones and listen to some of your favourite music.
   3. Tune in to the world! Notice five things around you that you weren’t aware of.
   4. Did not use coping strategies

IF RATED (a) OR (b) OR (c) TO THE ABOVE:

1. I found the strategy (specify) effective in coping with the voice/s.

IF RATED (d) TO THE ABOVE:

2. What was the reason you did not use any coping strategies?

- 1. I was too busy
  2. I didn’t think it would be helpful
  3. I was too distressed
  4. The voice/s weren’t bothering me enough
  5. I didn’t want to
  6. I forgot

*Daily coping and use of app*

1. Today, the app has helped me cope with the voices
2. Today, I have been feeling distressed by the voice/s
3. Today, it was hard for me to do things because of the voice/s
4. Today, I was distracted by the voice/s
5. Today, I noticed some things that made the voice/s more or less intense
6. Today, I did things to try to make the voice/s better
7. Today, I have been coping with the voice/s
